# On the use of Raman spectroscopy to characterize mass-produced graphene nanoplatelets

**DOI:** 10.3762/bjnano.14.42

**Published:** 2023-04-24

**Authors:** Keith R Paton, Konstantinos Despotelis, Naresh Kumar, Piers Turner, Andrew J Pollard

**Affiliations:** 1 National Physical Laboratory, Teddington, TW11 0LW, UK; 2 Department of Chemistry and Applied Biosciences, ETH Zurich, CH-8093 Zurich, Switzerland; 3 Department of Physics, University of Oxford, Oxford, UK

**Keywords:** few-layer graphene, graphene, metrology, quality control, Raman spectroscopy

## Abstract

Raman spectroscopy is one of the most common methods to characterize graphene-related 2D materials, providing information on a wide range of physical and chemical properties. Because of typical sample inhomogeneity, Raman spectra are acquired from several locations across a sample, and analysis is carried out on the averaged spectrum from all locations. This is then used to characterize the “quality” of the graphene produced, in particular the level of exfoliation for top-down manufactured materials. However, these have generally been developed using samples prepared with careful separation of unexfoliated materials. In this work we assess these metrics when applied to non-ideal samples, where unexfoliated graphite has been deliberately added to the exfoliated material. We demonstrate that previously published metrics, when applied to averaged spectra, do not allow the presence of this unexfoliated material to be reliably detected. Furthermore, when a sufficiently large number of spectra are acquired, it is found that by processing and classifying individual spectra, rather than the averaged spectrum, it is possible to identify the presence of this material in the sample, although quantification of the amount remains approximate. We therefore recommend this approach as a robust methodology for reliable characterization of mass-produced graphene-related 2D materials using confocal Raman spectroscopy.

## Introduction

Graphene and related 2D materials (GR2Ms) are now well established with commercial products available across a range of sectors, from sports and leisure products [1–2], through mobile phones [3] to automotive applications [4]. There are also a large number of producers of these materials [5], offering an array of products with a wide range of properties such as improved mechanical strength and higher thermal conductivity. To accelerate the further development and adoption of GR2Ms, it is critical to develop reliable and standardized methods to characterize the materials being produced and purchased. The publication of international standards is a key step in this process, such as recent publications on nomenclature [6] and structural characterization [7]. The measurement methods described in these standards, however, can be time-consuming and expensive. As the range of applications for GR2Ms expands, and with it the production volumes, there is an increasing need for faster methods that can be applied in-line or at-line. These quality control methods do not need the same level of accuracy and precision as those specified in international standards, but they do need to be validated against those methods. What is more important is repeatability and reproducibility, to allow for product monitoring over time. They also need to be able to provide results quickly, in a form that is easy to interpret, providing simple pass/fail outcomes.

Raman spectroscopy is one of the most widely used characterization tool for GR2Ms [8]. A search of Web of Science showed that of 97,532 articles published in the last five years with “Graphene” in the abstract, 9.3% also mentioned “Raman”. This is compared with atomic force microscopy (AFM) (2.4%), scanning electron microscopy (SEM) (11.4%), transmission electron microscopy (TEM) (7.2%) or X-ray photoelectron spectroscopy (XPS) (5.6%). It has the advantages of relatively low cost, simple sample preparation, quick measurements, and automated analysis, offering clear benefits for quality control applications. It has been demonstrated in several application areas as an in-line process analysis and control method [9–12].

Raman spectroscopy is particularly suited to the analysis of graphitic materials because of the large scattering cross section of graphitic materials and the large amount of information obtainable from a single measurement. For example, information on flake size, extent of structural defects, chemical or electronic doping, and strain and layer number can all be extracted from one spectrum [13–18]. As such, Raman spectroscopy is widely used by producers to assess the quality of their material, in particular the absence of graphite or nanoscale graphite. It is important to recall that graphene has been defined as a “single layer of carbon atoms with each atom bound to three neighbours in a honeycomb structure” with materials with more than one layer defined as *“*few-layer graphene” or “graphene nanoplatelets” [6]. This assessment is generally based on examining the shape of the so-called 2D peak (ca. 2700 cm^−1^), which, for Bernal stacking, shows clear changes on going from single-layer through few-layer graphene to graphite [19]. Bulk graphite typically shows a signal comprising two components, sometimes referred to as 2D_1_ and 2D_2_, with intensities approximately one fourth and half of that of the so-called G peak (ca. 1580 cm^−1^) [20]. In contrast, single-layer graphene typically yields a 2D peak comprising a single component, with an intensity of around double that of the G peak [19]. In between these two extremes, the peak shape evolves gradually, and while the 2D peak from bilayer graphene has been shown to comprise four components, deconvolution for higher layer numbers has not been reliably carried out. The spectrum recorded from flakes with ten or more layers is typically indistinguishable from that of bulk graphite. However, it is important to note that this behaviour can be affected by the stacking order. For example, for turbostratic graphite, where there is random rotational alignment between the layers, the 2D band also has the shape of a single Lorentzian line [21]. However, it typically has a larger width (45–60 cm^−1^) compared to single layer graphene (ca. 24 cm^−1^). The intensity of the peaks has also been shown to be influenced by the rotational angle in bilayer graphene, although the shape of the peak is largely unaffected [22]. Roscher et al. [23] have attempted to quantify the distinction between graphite and few-layer graphene based on the “goodness of fit” parameter when using a single Voigt function to fit the 2D peak. However, these changes in peak shape and (relative) intensity are generally only qualitative and have mostly been demonstrated only on well-defined materials, either from large mechanically exfoliated flakes or CVD-grown materials. When measuring the Raman spectrum from aggregated few-layer graphene (FLG) powder, where many particles are probed in a single measurement, the Raman 2D peak still typically appears as a symmetric shape, although with lower (relative) intensity and larger width than for single-layer graphene as a result of the convolution of many individual peaks [13].

It has previously been shown that Raman spectroscopy can be used to provide quantitative information on both flake thickness and lateral size of exfoliated graphene nanoplatelets (GNPs) [13]. These metrics are based on averaging spectra from multiple locations and using this averaged spectrum to make claims about the quality of the material. However, it is not clear how sensitive these metrics are to small amounts of thicker materials in a sample. Typically, the samples used to derive these metrics had also been carefully processed to ensure removal of unexfoliated graphite particles. In other cases, metrics are developed based on changes in Raman spectra with layer number from measurements on individual, well-defined, flakes that are not commercially produced [23]. Again, it is not clear if the same metrics can be applied to measurements performed on bulk material, where flakes are restacked or reaggregated. As many GNP products are produced through top-down manufacturing methods, which typically have a GNP yield of less than 100%, there is often a separation step in the production process [24–25]. Often based on a sedimentation process, this step removes the unexfoliated fraction of the material from the exfoliated product. However, if this process is not well designed and controlled, it is possible for the unwanted sediment to pass into the product stream. Due to this potential variation in material form within a sample, the results of any Raman analysis are typically based on averaging the spectra from a number of measurements across a sample. Although the sediment material has a distinct Raman spectrum compared to the commercially supplied GNP powder, it is not clear if Raman spectroscopy has the sensitivity to detect the presence of this material in the final product.

In this paper, we examine the effect that increasing amounts of unexfoliated graphitic material in a well-defined sample of GNPs have on the measured Raman spectra. First, a sample of GNP material is prepared through careful separation of unexfoliated material. Then, the observed changes for an averaged Raman spectrum are investigated while adding small amounts of graphite to the GNP sample. We then evaluate the ability of previously published metrics to identify the presence of this unexfoliated material in the GNP sample. Finally, we examine a more industrially relevant set of samples where fractions of the sediment removed during a separation stage are added back into the GNP sample. By examining the Raman spectrum averaged across many points on the sample, as well as individual spectra, the limits of the published metrics can be tested, and recommendations can be made for improved Raman analysis approaches.

## Methods

Rather than using commercial GNP products, we produced a dispersion by sonication-assisted liquid-phase exfoliation, using graphite (Sigma-Aldrich, UK, product no. 332461) and 1-methyl-2-pyrrolidone (NMP) (Sigma-Aldrich, UK, ACS Reagent, product number 443778) [26] as starting materials. An initial processing of the graphite was carried out to remove any impurities or small graphitic particles present in the material. To achieve this, graphite (0.8 g) was added to NMP (40 mL), and the mixture was sonicated at 20 kHz with a flat-head probe (130 W, CPX 130, Cole-Parmer Instruments, USA; 60% amplitude, 6 s on/2 s off cycle, 1 h sonication). The vessel was kept cool by immersing it in an ice bath during processing. The dispersion was then centrifuged at 5000*g* for 1 h, and the supernatant was separated from the sediment and discarded. Fresh NMP was added to bring the volume up to 40 mL, and the mixture was returned to the sonic tip. Using the same conditions as for the initial processing step, the mixture was sonicated for 5 h to exfoliate the graphite.

To ensure that thicker material was removed from the dispersion, an abbreviated cascade centrifugation process was applied [27]. The dispersion obtained following 5 h of sonication was centrifuged at low speed (250*g*) for 2 h to remove the very largest particles of unexfoliated graphite. The supernatant from this step was then centrifuged at 1000*g* for 2 h to sediment the larger particles of GNPs. The supernatant from this step was then further centrifuged at 5000*g* for 2 h to sediment the thinner GNPs. This sediment was then mixed with fresh NMP (50 mL), and the mixture was vortex-mixed briefly and subsequently sonicated in a bath sonicator for 5 min to re-disperse the sediment. This sample is referred to as “GNP_ref_”.

The concentration of GNP_ref_ was measured using UV–vis extinction spectroscopy (Perkin-Elmer 850, PerkinElmer, UK), using a cuvette with 10 mm path length. Measuring the extinction at 660 nm and using an extinction coefficient of 4237 mL·mg^−1^·m^−1^ [28] yielded a concentration of 0.028 mg·mL^−1^.

To characterize the thickness of the particles in GNP_ref_, the dispersion was drop-cast on to a cleaned Si/SiO_2_ (300 nm thick oxide layer) wafer. Before deposition, the dispersion was diluted by a factor of 10 in fresh NMP. 10 μL of the diluted dispersion was then drop-cast on a Si/SiO_2_ wafer at a temperature of 200 °C. To remove residual NMP, the sample was dried overnight in a vacuum oven at 60 °C.

AFM measurements of the deposited flakes were carried out using Cypher AFM (Asylum Research, Oxford Instruments, UK). AFM images were recorded using Si AFM probes (MikroMasch HQ:NSC15, 40 N/m, 325 kHz, MikroMasch, Bulgaria) in tapping-mode feedback. AFM images were measured in square areas between 6 μm × 6 μm and 8 μm × 8 μm using 1024 × 1024 pixels with a scan speed below 20 μm·s^−1^.

To prepare the mixed GNP_ref_/graphite samples, 1.2 mg of the as-purchased graphite was mixed with 40 mL of NMP, and the mixture bath was sonicated for 30 min. Sonication was carried out to reduce the particle size while still maintaining the thickness to be graphite-like [29]. The graphite and GNP_ref_ dispersions were then mixed to obtain graphite mass fractions as given in Table 1.

**Table 1:** Graphite content in the samples measured in this study.

Sample #	GNP_ref_/wt %	Graphite/wt %

1	0	100
2	90	10
3	95	5
4	98	2
5	99	1
6	99.5	0.5
7	100	0

To prepare the mixed GNP_ref_/sediment samples, a fresh GNP_ref_ sample was prepared by sonication as described above. After the initial centrifugation step at 250*g*, however, the sediment was retained and redispersed in fresh NMP. The concentration of the resulting dispersion was measured using UV–vis spectroscopy, as described above. The dispersion was then added to the GNP_ref_ sample to produce 13 mixed GNP/sediment samples with sediment concentrations of 0.5, 1, 2, 5, 10, 25, 35, 50, 65, 75, and 90 wt %.

Each mixed dispersion was then vacuum-filtered through alumina membranes (20 nm pore size), rinsed with IPA to remove residual NMP, and dried in a vacuum oven at 60 °C overnight. For samples 2 to 7, 3 mL of the dispersion was filtered, while for sample 1, ca. 30 mL was used to ensure adequate coverage of the membrane.

Raman spectra of the filtered films on the membrane were recorded using a Renishaw Qontor confocal spectrometer (Renishaw plc., UK) using a 532 nm excitation laser and a 2400 L/mm grating. An area of 20 μm × 20 μm of the film was mapped, with 1 μm distance between measurement locations. Spectra were recorded between 1000 cm^−1^ and 3000 cm^−1^ Raman shift, using 5% of the maximum power (ca. 0.8 mW incident on the sample), 10 s acquisition time, and a 100× (0.9 NA) objective lens.

Spectra were processed to remove cosmic ray artefacts, and a baseline was subtracted using the “Intelligent Fitting” algorithm in the Wire 5.4 software (Renishaw plc., UK) based on an 11-point polynomial. Each spectrum was then normalised to give intensities between 0 and 1, and the spectra from each map were averaged. D band, G band, D’ band, and 2D band of all spectra, either individual or averaged, were fitted using Lorenztian functions. The peaks were fitted together, with an offset baseline. We have averaged across 441 points in a sample, which is more than is used in a typical workflow, and the effect of the number of points measured is examined later in this paper.

## Results and Discussion

### AFM results

**Figure 1:**
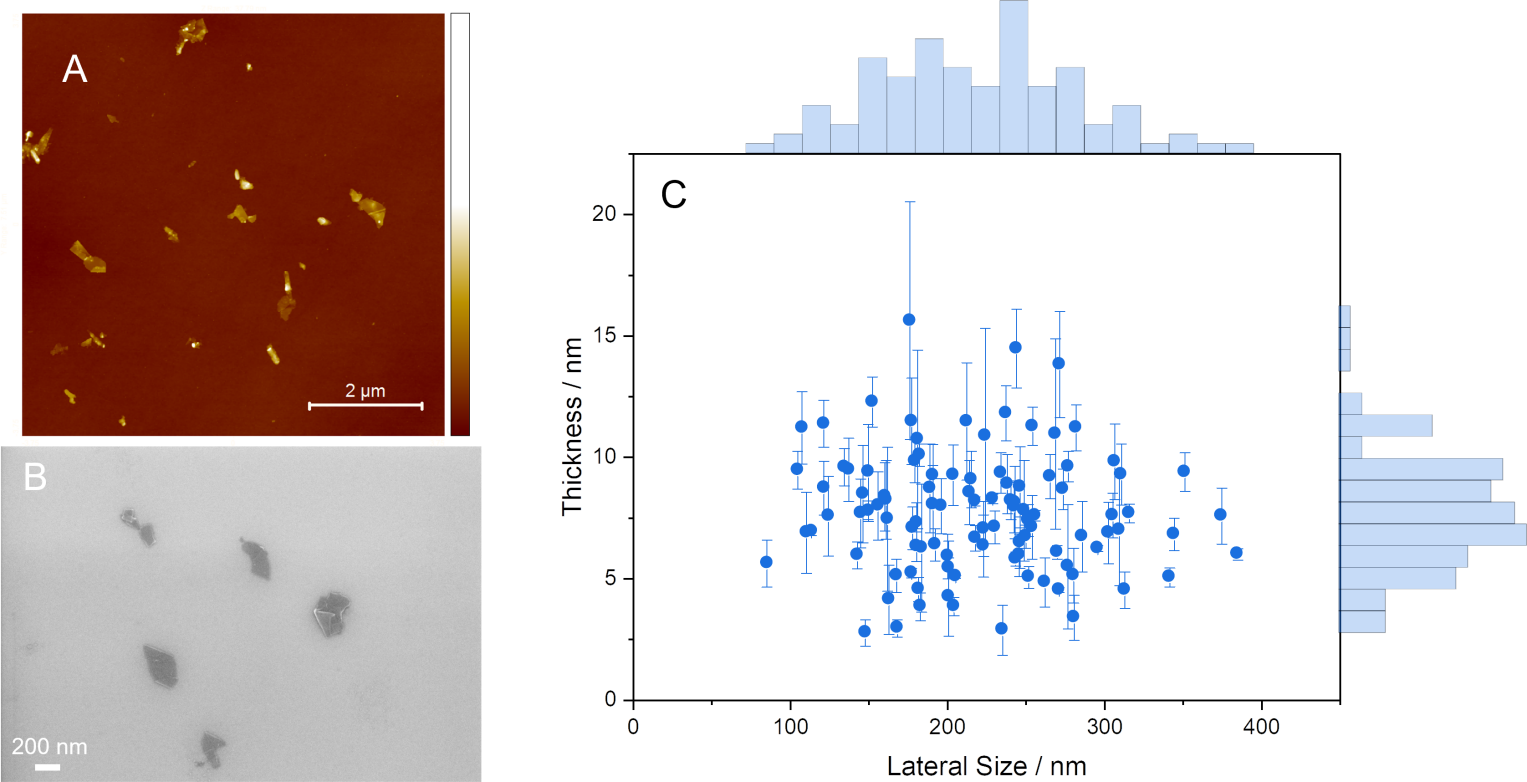
(A) Example AFM image of flakes from the GNP_ref_ sample; the scale bar is 2 μm. (B) Example SEM image of flakes from the GNP_ref_ sample; the scale bar is 200 nm. (C) Flake sizes of GNP_ref_ sample measured by AFM with histograms of the lateral size and thickness distributions.

The aim of the sample preparation protocol for GNP_ref_ is to obtain a dispersion that contains primarily graphene nanoplatelets [6] without unexfoliated graphite particles. To evaluate this [30–32], AFM was carried out to measure the thickness of the flakes from the dispersion. A representative AFM image of the flakes contained in the GNP dispersion is presented in Figure 1. The measurements were carried out according to ISO TS 21356-1 [7].

The height of the flakes ranged from 2.8 to 15.6 nm, with 85.3% of the measured flakes thinner than 10 nm and 3.9% of the flakes less than 3.4 nm thick. These are particles that can be classified as FLG in thickness [6]. We note that this is a higher content of FLG than in many such powders on the market [33]. The flakes had a mean height of 7.7 ± 2.5 nm (mean ± standard deviation) and a median height of 7.6 nm. A scatter plot showing the correlation between the flake height (i.e., thickness) and their lateral size is shown in Figure 1c. Figure 1c indicates that the height of the flakes in the GNP dispersion is independent of the lateral size. The lateral size of the measured flakes ranged from 85 nm to 385 nm, with a mean lateral size of 219 ± 64 nm and a median lateral size of 218 nm. We did not attempt to give a number of layers for these flakes. Yet, we do note that while the natural interlayer spacing for graphite is 0.34 nm, it has been reported previously that for similarly produced flakes, monolayer flakes had a measured thickness of 2 nm, with each additional monolayer adding 0.95 nm to the thickness [25].

### Graphite addition

#### Raman spectroscopy results

**Figure 2:**
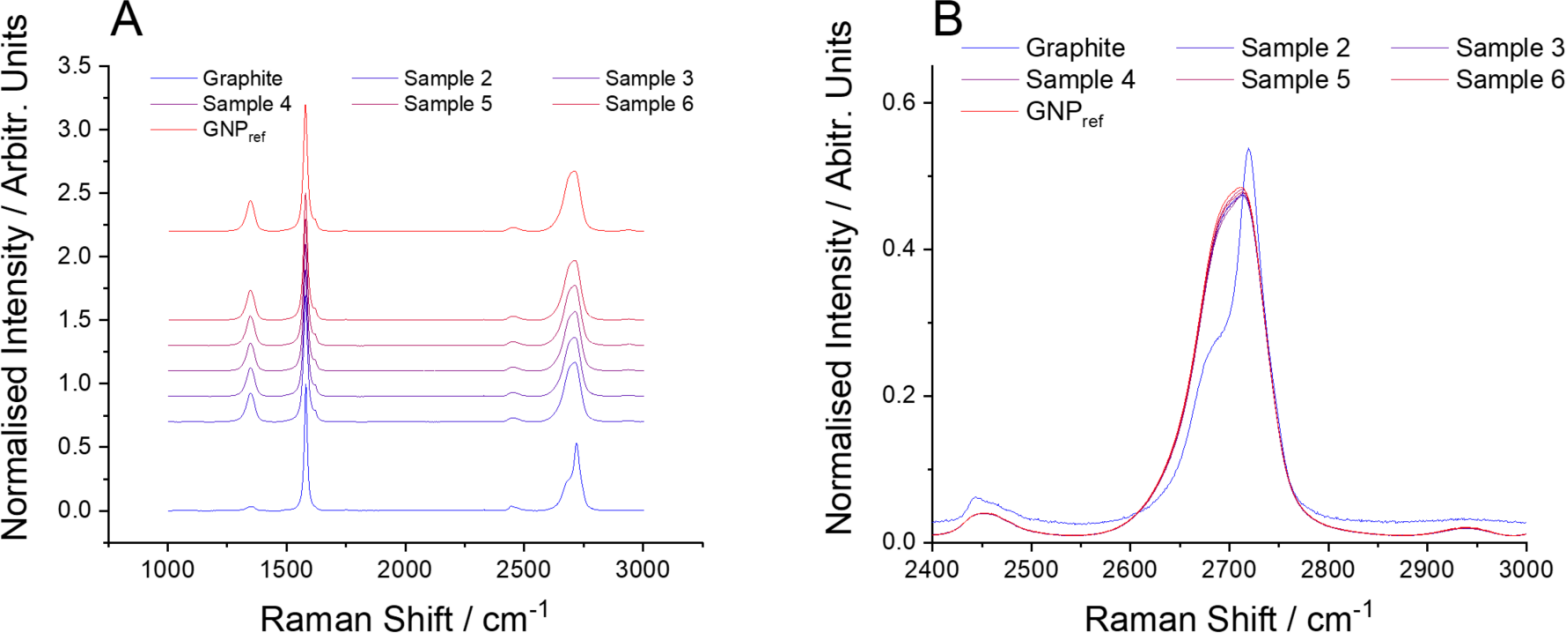
(A) Averaged spectra from samples of GNP_ref_ with additions of graphite. The bottom spectrum (blue) is from graphite only. The top spectrum (red) is from GNP_ref_ only. All other spectra are mixtures of GNP_ref_ and graphite. (B) Overlaid spectra showing the region of the 2D peak.

Samples of GNP_ref_ with different amounts of added graphite were analysed with Raman spectroscopy. As shown in Figure 2, the spectrum measured from pure graphite is distinct from that of the GNP_ref_ with a lower D band intensity (ca. 1350 cm^−1^) and a distinct shoulder on the 2D band at ca. 2700 cm^−1^. In contrast, there is no clear difference in the average spectra recorded in any of samples 2–7. All of them are almost identical to the spectrum of the GNP_ref_ sample. In other words, despite the samples contained up to 10 wt % graphite, a measurement protocol that might be considered typical yields a spectrum that is almost indistinguishable from that of graphene nanoplatelets.

**Figure 3:**
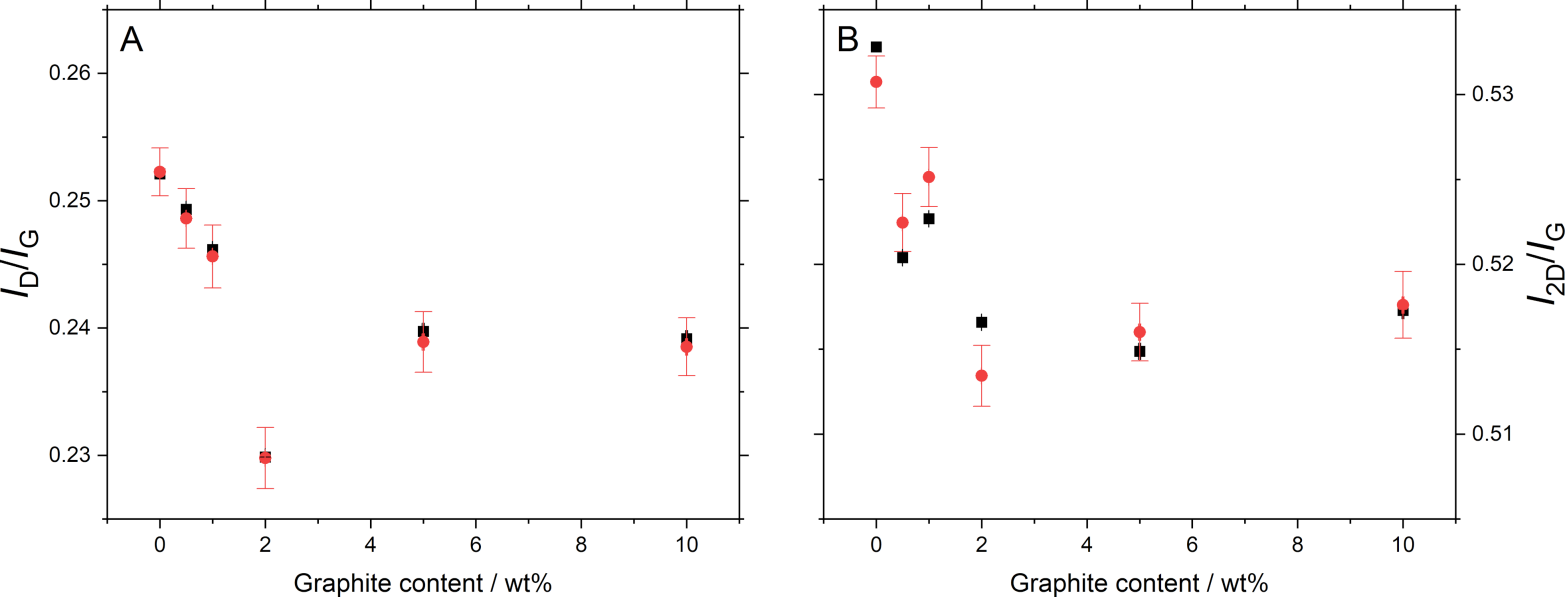
Fitted peak intensity ratios. (A) *I*_D_/*I*_G_ and (B) *I*_2D_/*I*_G_, showing the values from the averaged spectra (black) with the combined standard error of the fit, and the mean value of the fits across each map (red) with the standard error of the mean.

The averaged spectra were fitted to obtain the peak intensity ratios, as shown in Figure 3. To investigate any differences in the measured spectra, each spectrum in each map was also fitted to obtain the peak intensities of the D peak (1350 cm^−1^), the G peak (1580 cm^−1^), the D’ peak (1620 cm^−1^), and the 2D peak (2700 cm^−1^) (see Supporting Information File 1, Figure S1 for distributions of *I*_D_/*I*_G_ values). The median of the values was then calculated, together with the standard error of the mean. Note that the 2D peak has not been fitted for the graphite sample as the 2D peak in the graphite spectrum is a poor fit to a single Lorentzian function. For all other spectra, a single peak was used to fit the 2D band. The intensity ratio between D peak and G peak (*I*_D_/*I*_G_) has been shown to correlate with the lateral sizes of exfoliated flakes [30–32], while the intensity ratio between 2D peak and G peak (*I*_2D_/*I*_G_) varies with flake thickness [8,19,34]. As shown in Figure 3, in both cases (averaged (black) and individual (red)), there is a fall in the value of *I*_D_/*I*_G_ with increasing graphite content. This would be expected as the graphite particles have a larger lateral size compared to the exfoliated GNPs. However, this trend is only seen up to 2 wt % graphite, with no further change observed above this graphite weight percentage value. The overall variation in *I*_D_/*I*_G_ ratio between samples is also relatively small. This trend is matched by the *I*_2D_/*I*_G_ ratio, which again would be expected due to the thicker particles in the graphite materials. See Supporting Information File 1, Figure S2 for plots including the 100 wt % graphite sample.

**Figure 4:**
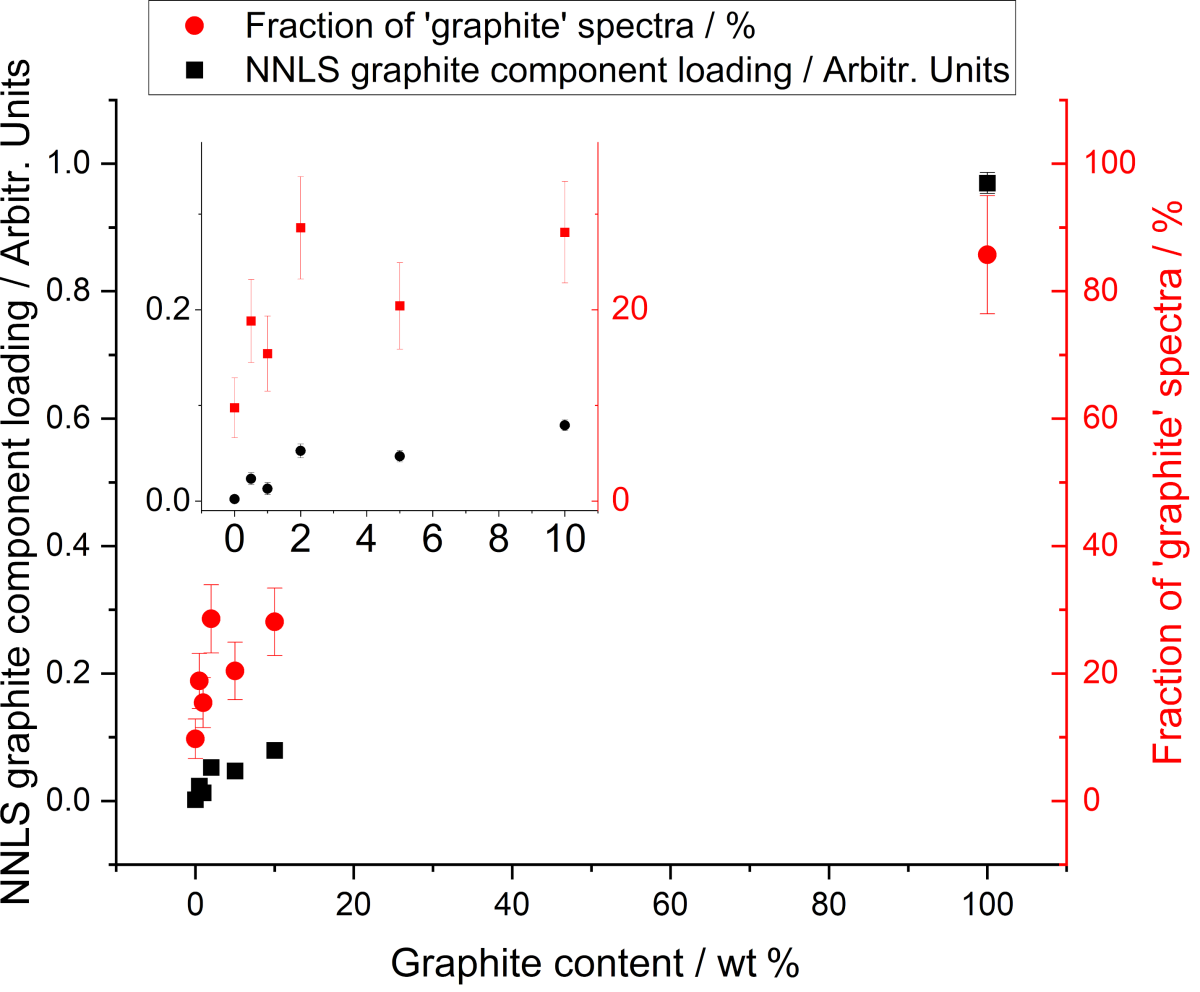
Median value of the graphite component loading value, as determined by a non-negative least-squares fit (black squares, left axis) and the fraction of points that show a graphite-like spectrum (red squares, right axis). A graphite-like spectrum is defined as having a correlation value to the graphite spectrum of greater than 0.15, as obtained from a non-negative linear least-squares procedure when fitted with both a GNP and graphite spectrum. The inset shows the low-loading region of the graph, with the same axes.

While metrics such as mean intensity ratios are widely used, a more reliable identifier to distinguish graphite from graphene/few-layer graphene is the shape of the 2D peak. For graphite, it shows a clear shoulder on the low-wavenumber side of the peak, as seen in Figure 2b, and is therefore best fitted with two individual Lorentzian peaks. In order to investigate whether Raman spectroscopy can be used to quantify the proportion of graphite in a bulk sample such as this, a non-negative linear least-squares (NNLS) algorithm [35] has been applied to the spectra to calculate the quality of the match to either a graphite or GNP Raman spectrum. This fitting was carried out using the “Component Analysis” tool in Wire 5.4, using the average spectra from graphite and GNP samples respectively as the “pure” components. No additional baseline or normalization was applied during the analysis, and the spectra were fitted directly (rather than a derivative of the spectra). Similar fitting can be implemented in a range of other analysis packages. A higher value of the correlation value indicates a better match to that component. Plotting the median value of the graphite correlation value (Figure 4, black data points) shows that it increases with increasing graphite content across the full range of measured graphite loading. This approach appears therefore capable of discerning the amount of graphite in a sample.

From the distribution of component loadings from the GNP_ref_ sample (see Supporting Information File 1, Figure S2) we can define a threshold value for a spectrum that corresponds to graphite. This is taken as the *d*_90_ value from the distribution. Based on this, we can then classify each pixel measured across a sample as either “graphite” or “GNP”. From this, we can calculate a fraction of graphite in the sample, as shown in Figure 4 (red data points). The measured fraction of graphite-like spectra increases with nominal graphite content in the sample. The fraction for the graphite sample is close to 100% confirming that the sample has very high levels of thick, graphite-like flakes (see Supporting Information File 1, Figure S3 for plots of other metrics shown to include the 100% graphite sample).

The analysis based on simple peak fitting of the spectra measured from samples with graphite added to GNP_ref_ have shown that there is very limited ability to reliably identify the presence of graphite. In contrast, applying a least-squares fitting process to estimate the graphite loading in each sample shows better ability to identify those additions. However, it is of interest to examine how previously published metrics perform on the same samples.

#### Comparison to literature metrics

As mentioned above, several metrics have previously been published to attempt to obtain quantitative information of the flake thickness from Raman measurements. A selection of these have been applied to the data acquired as part of this work to evaluate whether these metrics can be implemented successfully. Two metrics have been presented by Backes et al. [13] to quantify the mean number of layers from Raman spectra measurements. The first of these (M_1_) is based on the *I*_2D_/*I*_G_ ratio as calculated above, and an empirical fit to the mean number of layers *N* was found as


[1] $$\langle N\rangle =1.04*{\left(I_{2\text{D}}/I_{\text{G}}\right)}^{-2.32}.$$

The second metric (M_2_) is based on the ratio of intensities at two different locations in the 2D band [13]. The first location is that of the maximum of the 2D band of the parent graphite material. In this case, this is the 100% graphite sample and the location is ω_1_ = 2719 cm^−1^. The second location is 30 cm^−1^ below the first location, that is, ω_2_ = 2689 cm^−1^. The ratio of the intensities at these two locations is then normalised to the ratio from the parent graphite, such that


[2] $${\text{M}}_{2}={\left(\frac{I_{\omega_{1}}}{I_{\omega_{2}}}\right)}_{\text{GNP}}/{\left(\frac{I_{\omega_{1}}}{I_{\omega_{2}}}\right)}_{\text{G'ite}}.$$

The mean number of layers per flake can then be calculated according to


[3] $$\langle N\rangle =0.83e^{3.6{\text{M}}_{2}}.$$

An alternative approach has been proposed, based on the change in shape of the 2D band as the number of layers increases [23,36–38]. It is known that Raman measurements of single-layer graphene produces a single peak, whereas graphite produces a bimodal peak [19]. Furthermore, it has also been shown that the peak shape changes gradually between these two extreme cases [34]. As a simple way to characterize this change, the 2D peak can be fitted to a single Voigt peak, and the quality of the fit, as quantified by the *R*^2^ value, can be correlated to the mean number of layers in the sample. Results from the Casiraghi group [39] suggest that a single-layer flake has *R*^2^ > 0.987 and few-layer graphene has 0.987 > *R*^2^ > 0.985. *R*^2^ values less than 0.985 would indicate a thicker flake with more than seven layers.

**Figure 5:**
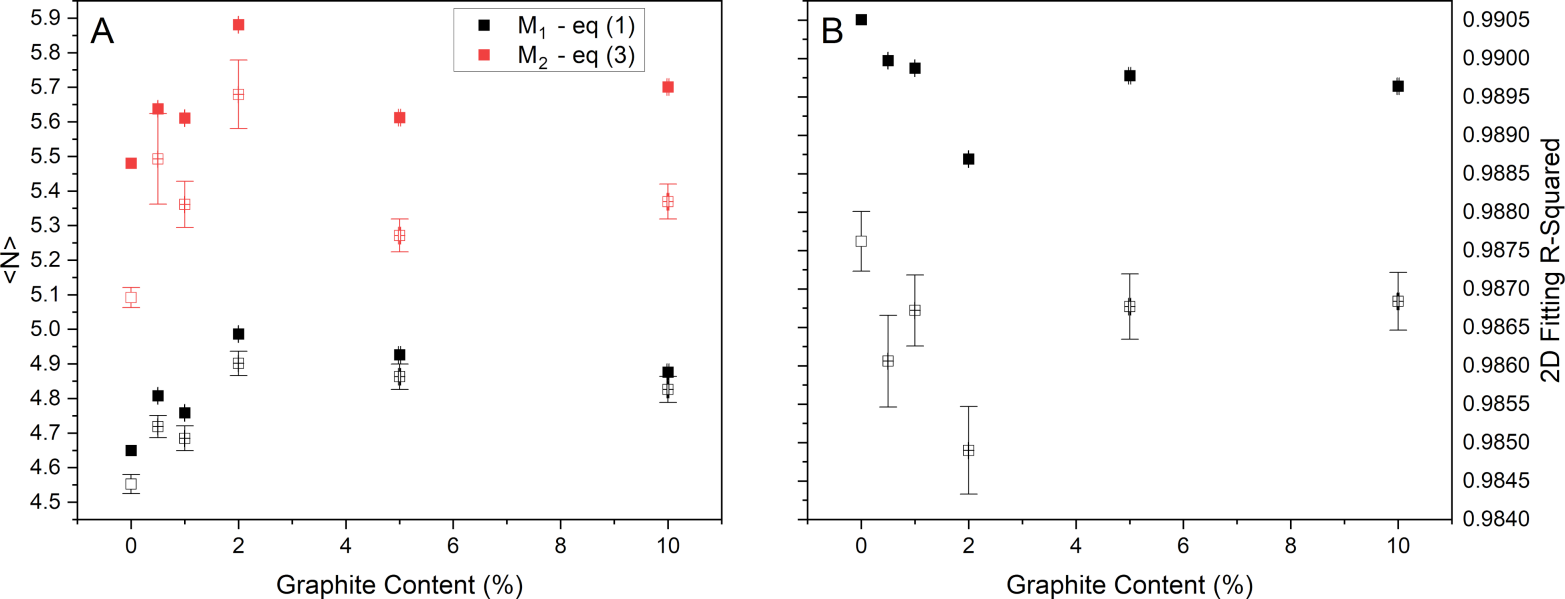
Comparison of literature metrics applied to the current data. (A) Mean number of layers calculated for the two metrics published by Backes and co-workers [13]. (B) *R*^2^ value for fitting the 2D peak, as proposed by Roscher and co-workers [23]. In both cases, results are shown from analysis of both averaged spectra (filled markers) and individual spectra within each map (open markers). In the latter case, the median value is shown with the error bars showing the standard deviation.

We have applied the three metrics described above to the current data, both the averaged spectra and each individual spectrum, with the results shown in Figure 5. It is clear that, neither of the simple peak intensity ratios described above are effective at discriminating the presence of graphite within the sample. Some changes are seen up to 2 wt % graphite, but not beyond this (see Supporting Information File 1, Figure S3 for plots including the 100 wt % graphite sample). We also note that the number of layers for the GNP_ref_ sample predicted by the M_1_ and M_2_ metrics are in reasonable agreement with the AFM results presented in Figure 1.

The metric based on the 2D peak shape was derived from measurements on single flakes with well-characterized thicknesses. It is known that when measuring bulk, reaggregated flakes, the 2D peak retains a symmetric shape, even to values of mean thickness where a single flake of equivalent thickness would yield a clear shoulder in the Raman spectrum [13]. It is clear from these measurements that this method also does not have the sensitivity to identify the presence of small amounts of graphite in a sample. Similarly, although the metrics from Backes et al. were both derived from measurements of reaggregated materials, they are also unable to identify the graphite content. The samples used to develop these methods, however, were carefully prepared to exclude all graphite-like material, which may not be representative of commercially produced products. It is of particular note that the M_2_ and *R*^2^ metrics give significantly different values of mean layer number depending on whether the single averaged spectrum or the individual spectra are analysed. It is clear that the sequence of analysis (analysing an averaged spectrum vs averaging values from individual spectra) can have an effect on the results obtained for these methods. In the case of the *R*^2^-based metric, this difference is likely to be a result of the reduction in noise level affecting the value of *R*^2^ (see Supporting Information File 1). Additional metrics, including G peak width and the correlation between peak area ratios and G peak width, show similar trends (see Supporting Information File 1).

### Sediment additions

While the results presented above demonstrate the limitations of Raman spectroscopy to identify the presence of graphite in a GNP sample, of more relevance is the question of identifying unexfoliated sediment. In typical top-down exfoliation processes, the yield of few-layer graphene or graphene nanoplatelets is very small, often less than 1 wt %. Hence, there is a need to separate this product from the processed but unexfoliated material (which can often be recycled through the process again). Often this is accomplished through a centrifuge-based method, hence this material is referred to as sediment here. In order to maximise production yield, there is a need to maximise the amount of GNP material extracted, while to maintain product quality, it is important to prevent sediment accidentally ending up in the extracted GNP fraction. To allow for a commercially viable, industrial scale-up, it is important to understand the performance of this separation step in order to minimise processing time and to maximise separation efficiency.

**Figure 6:**
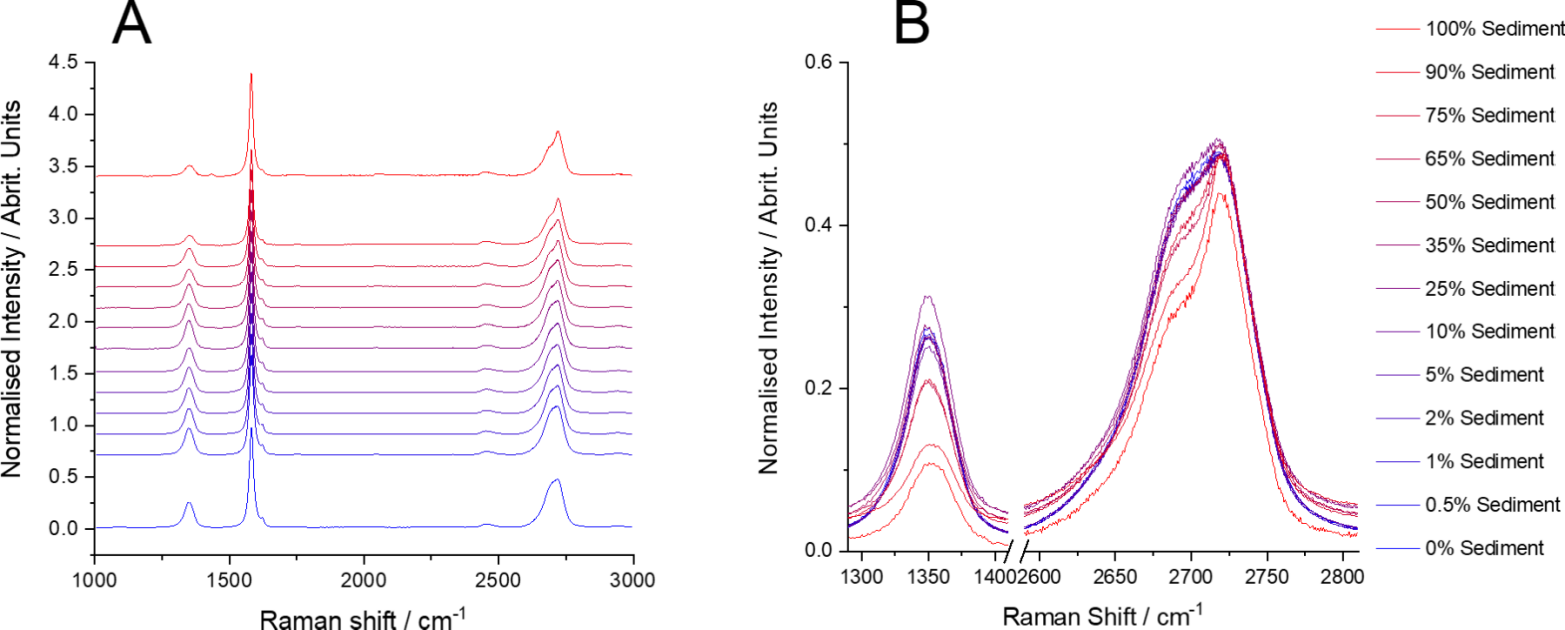
(A) Averaged spectra from samples of GNP_ref_ with additions of sediment. Bottom spectrum (blue) is sediment only. Top spectrum (red) is GNP_ref_ only. All other spectra are mixtures of GNP_ref_ and sediment. (B) Overlaid spectra showing the region of the D peak and the 2D peak.

To investigate if Raman spectroscopy can be used for this purpose, a fresh sample of GNP_ref_ was prepared as described above, using the same processing conditions. Instead of fresh graphite however, the sediment from the first, low-speed centrifugation step (at 250*g*) was recovered, and re-mixed into the GNP_ref_ sample. These mixed samples were then filtered and measured with Raman spectroscopy using the same settings described above. The spectra measured by averaging across the mapped area are shown in Figure 6, where it can be seen that up to ca. 50 wt % sediment, there is little change between them. This is despite the fact that the sediment spectrum is clearly different and closely matches what would be expected from graphite.

**Figure 7:**
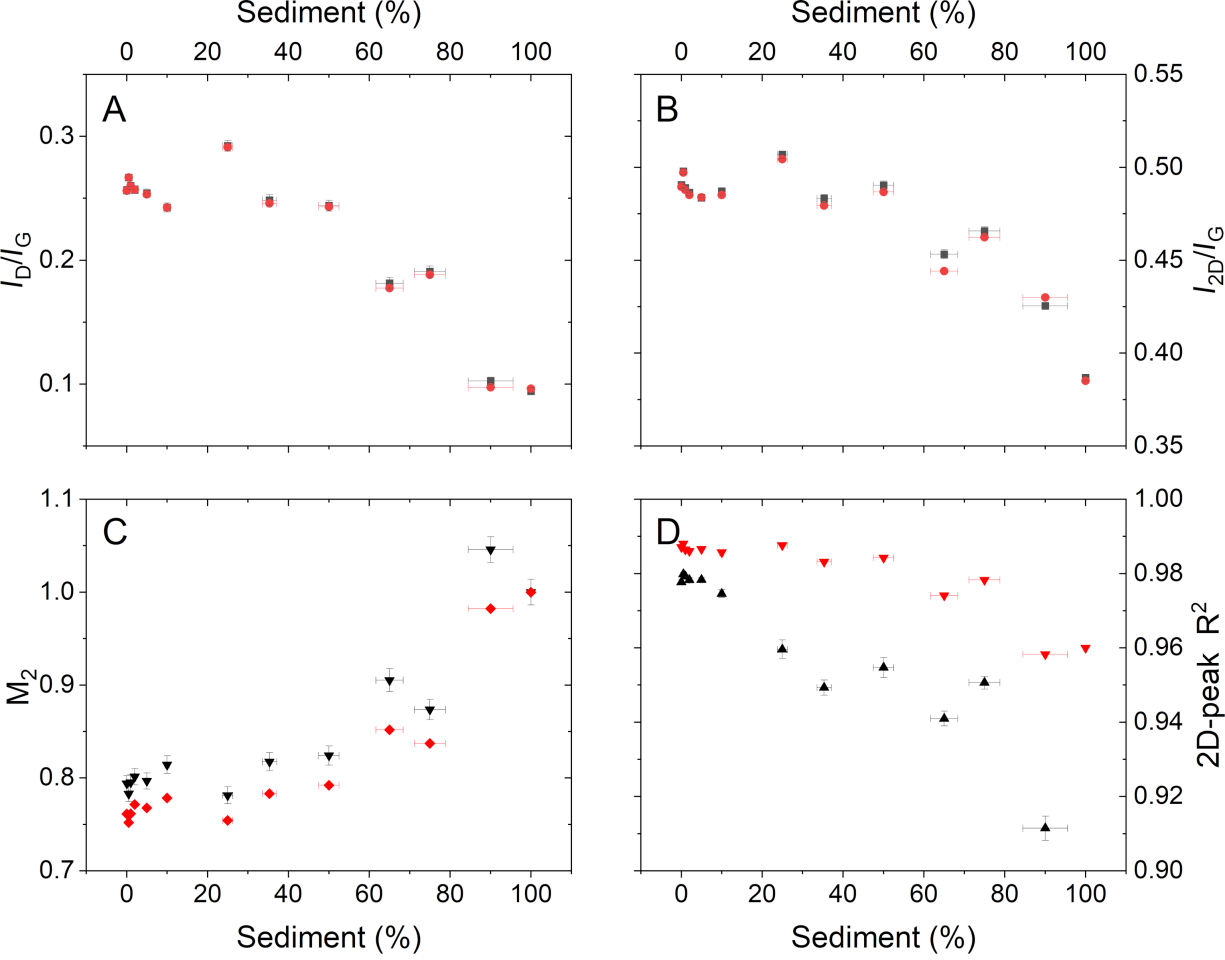
Values of the metrics at each sediment loading. (A) *I*_D_/*I*_G_ intensity ratio; (B) *I*_2D_/*I*_G_ intensity ratio; (C) M_2_ metric from Backes et al. [13]; (D) *R*^2^ value from fitting the 2D peak to a single Voigt function. Results are shown from both the average spectra (red) and the mean value from the fitting of each spectrum in a map (black) showing the standard deviation as the error bar.

This result confirms that simple inspection of a Raman spectrum, even when averaged across a large number of locations in a sample, cannot be relied on to confirm the absence of unexfoliated graphite material. Fitting these averaged spectra confirms that the simple intensity ratios (Figure 7a,b) do not show any significant differences up to between 35 wt % and 50 wt % sediment added. This is also true of the metric M_2_ (Figure 7c) and the *R*^2^ value (Figure 7d) from fitting the 2D peak to a single Voigt function.

The results in Figure 7 suggest that using a simple metric based on peak or spectral intensities is not useful to identify the presence of up to 20 wt % unexfoliated sediment in a sample of GNPs. This is the case both when analysing an averaged spectrum or when averaging the values obtained from individual spectra across the map. Of the possible metrics examined here, the method based on the *R*^2^ value from fitting the 2D peak shows the widest range of use when many individual Raman spectra are fitted and then averaged, as shown in Figure 7D. However, even in this case there is little change in the calculated value from samples with up to 10 wt % sediment added.

**Figure 8:**
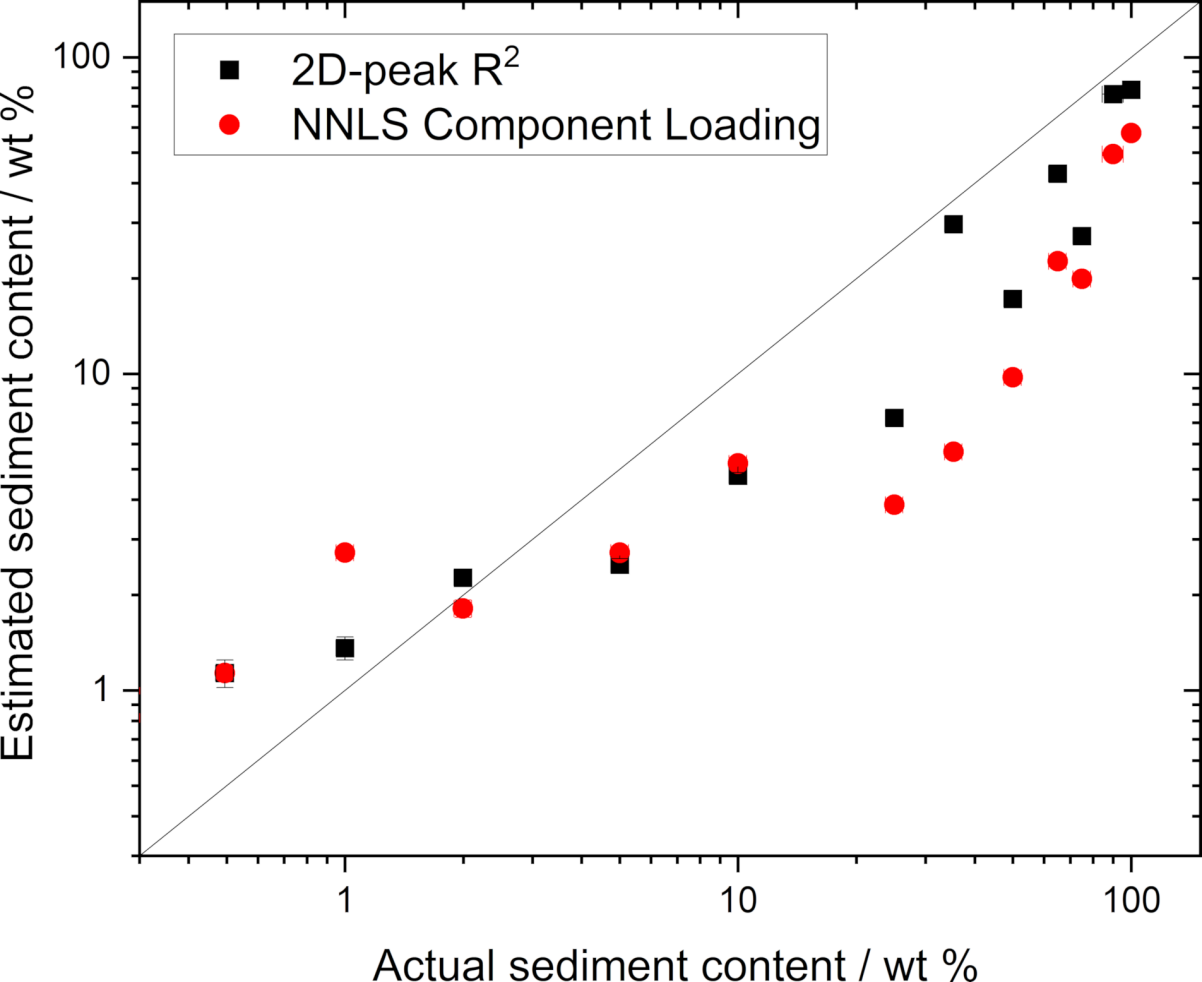
Estimated sediment content calculated from the fraction of measured spectra that are “sediment-like” based on either adjusted *R*^2^ value from fitting the 2D peak or from NNLS fitting. The line shows the expected linear trend, rather than a fit to the data.

Using a value averaged across a map, with appropriate uncertainties, is useful and easy to interpret. However, information is lost in the process of averaging, even when applying the metrics to individual spectra. An alternative approach may be to apply a classification of each spectrum and then to calculate the fractions that are “sediment-like”. This can be done in two ways: first, by adopting the approach of Roscher et al. [23] and defining a cut-off in the *R*^2^ value from fitting the 2D peak and, second, by running a non-negative least squares fit and defining a cut-off for the “sediment-like” spectral loading, as implemented above for graphite additions. The value of the threshold has here been set to minimise the mean-squared variance between the calculated fraction and the known loading of sediment material. The estimated sediment content based on the fraction of “sediment-like” spectra for each sample is shown in Figure 8.

It can be seen that if the individual spectra in the map are classified as “sediment” or “GNP” without any averaging, the presence of this non-GNP material can be identified more reliably than when using averaged values. We have calculated the variance from the nominal loading according to:


[4] $$\frac{\left|f_{\text{pred}}-f_{\text{nom}}\right|}{f_{\text{nom}}}\times 100,$$

where *f*_pred_ is the fraction of sediment predicted by the metric, and *f*_nom_ is the weight fraction of added sediment. The largest variation is still seen at the lowest loading of sediment. Using a threshold value of 1.09 × 10^−3^, the mean absolute deviation from the expected value is 73% with a maximum of 172% for 1 wt % using the NNLS fitting. Using the *R*^2^ value from the 2D peak fit with a threshold of 0.949 gives a mean absolute deviation of 47%, with a maximum of 129% for the 0.5 wt % sample. Tables with the full values of the predicted sediment content and the variance and the ratio between nominal and predicted values are provided in Supporting Information File 1, Table S1 and Table S2. It is a useful finding that the mean deviation is lower for the *R*^2^ approach as this approach is significantly easier to implement computationally and the spectra of the pure components are not required. This ease of implementation is critical when designing a quality control system. However, it is important to keep in mind that the value of *R^2^* obtained is affected by the signal-to-noise ratio of the spectrum (see Supporting Information File 1, Figure S4 and Figure S5). We therefore do not intend to suggest that the values of the thresholds given here are universal values.

**Figure 9:**
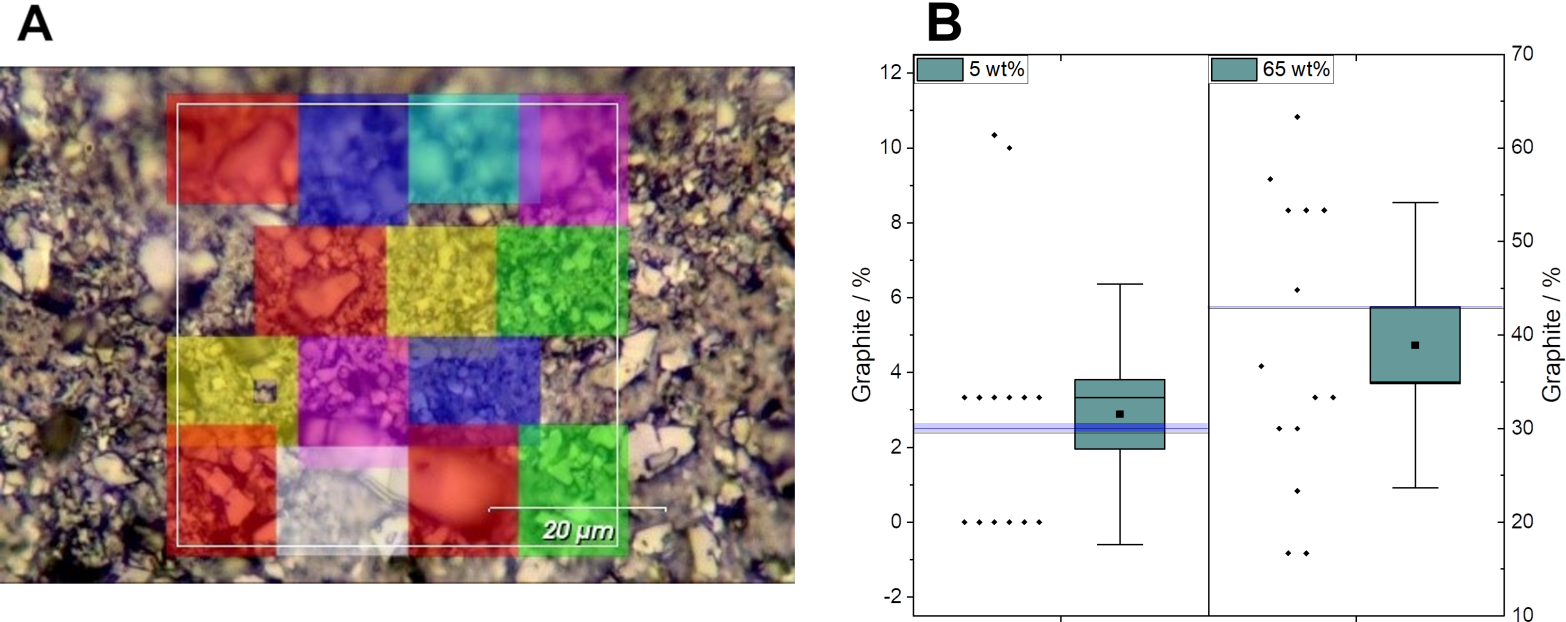
(A) Optical image of mapped area of sample with 65 wt % sediment added. Each coloured box shows a sub-map of 30 points taken from the full map of 441 points, indicated by the white-bordered box. (B) Calculated percentage of graphite for samples with 5 and 65 wt % sediment, based on the *R*^2^ value from fitting the 2D peak. Each box shows the standard error of the mean, with the whiskers showing one standard deviation. The square marker indicates the mean value, while the light line across each box shows the median. The thick line across each panel shows the average value from the full map area. The individual data points are shown to the left of each box, off-set in the *x*-direction for clarity.

It is important to consider that the sediment component is present in discrete particles, which are significantly larger than the GNP particles and generally larger than the laser spot size (above 1 μm with 100× objective lens). Due to the larger size and thickness of the sediment particles compared to the GNP particles, a given weight fraction of sediment will correspond to a significantly lower number fraction of particles [40]. This difference will be more pronounced at low mass loading of sediment, where a small number of particles will be needed to provide the required mass. This increases the likelihood that the area mapped does not contain a representative number of sediment particles and may therefore skew the results. To investigate this effect, we have taken the samples with 5 wt % and 65 wt % sediment, and calculated the metrics obtained from smaller sub-maps, as shown in Figure 9. Figure 9A shows the white-light image of the sample with 65 wt % sediment addition, with the sub-maps used overlaid.

When taking 14 sub-maps of 30 points and applying the criteria for “sediment-like” spectrum defined above for the *R*^2^ value of 2D peak fitting, the fraction of sediment measured varied from 0% to 10.3%, with a mean value of 2.9% ± 0.2%, with a standard deviation of 3.5%. Similarly, for the sample with 65 wt % sediment, the values range from 16.7% to 63.3%, with a mean value of 38.9% ± 1.1% and standard deviation of 15.3%. It is therefore clear that if only a small number of points are measured, it is possible to record a significant variation in the measured concentration of sediment in a sample. Indeed, for the 5 wt % GNP_ref_ sample, six of the sub-map analyses would indicate that there is no sediment present. These results emphasise the importance of acquiring a large number of spectra from a sufficiently wide area of the sample to ensure representative sampling. This is in line with Coleman et al. [41] who have previously discussed the issue of sampling.

## Conclusion

While Raman spectroscopy is a powerful and widely used technique to characterize graphene-related 2D materials, this work shows that care is needed when interpreting measurements from bulk samples containing many particles. Measurements on individual graphitic particles can distinguish between graphite, few-layer graphene, and graphene. However, measurements on mass-produced samples can be more difficult to interpret. In this case, which is more similar to the requirements of a quality control process for industrially produced powders containing GNPs, each measurement location will be sampling multiple particles, which will have different number of layers and lateral sizes.

We have shown in this work that when measuring samples with significant fractions of unexfoliated material, quantification can be difficult. When analysing the average spectum from a large number of locations in a bulk sample, the presence of up to 10 wt % graphite can not be reliably identified. When adding processed graphite (“sediment”) into samples, this limit is extended up to 50 wt % when analysing the averaged spectrum. However, we have shown that if individual measurement locations are analysed and classified separately, it is possible to identify the presence of this material in the sample, although quantification of the amount remains approximate. As has been shown previously, for this to be reliable, a large number of spectra need to be measured to ensure representative sampling of the material.

In light of these results, it is suggested that while Raman spectroscopy remains a powerful tool, in order to reliably identify the presence of graphite material in a GNP sample, individual spectra need to be analysed and classified before averaging. Using a metric based around the residual of a simple peak fitting means that this can be implemented in an automated, unsupervised method. This is essential for the use in a quality control process for industrial production of materials.

## Supporting Information

File 1 Additional experimental data.

## References

[1] (2023). Inoveight Limited Inov-8 Unveil World's First-Ever Graphene Sports Shoes, 2018.

[2] (2023). Vittoria S.p.A. Bicycle tires - powered by graphene (2017).

[3] (2023). Huawei Technologies Co. Ltd. Huawei Achieves Major Breakthrough in Graphene-Assisted High Temperature Li-ion Batteries (2016).

[4] (2023). The Ford Motor Company Cell phones, sporting goods, and soon, cars: Ford innovates with "miracle" material, powerful graphene for vehicle parts (2018).

[5] SIO Grafen, Graphene Supplier Guide.

[6] (2023). ISO, Nanotechnologies — Vocabulary. In Part 13: Graphene and related two-dimensional (2D) materials, 2016; Vol. ISO/TS 80004-13:2017.

[7] (2023). Nanotechnologies — Structural characterization of graphene. In Part 1: Graphene from powders and dispersions, 2021; Vol. ISO/TS 21356-1:2021(E).

[8] Ferrari A C, Basko D M (2013). Nat Nanotechnol.

[9] Févotte G (2007). Chem Eng Res Des.

[10] Echtermeyer A, Marks C, Mitsos A, Viell J (2021). Appl Spectrosc.

[11] Harting J, Kleinebudde P (2018). Eur J Pharm Biopharm.

[12] Harms Z D, Shi Z, Kulkarni R A, Myers D P (2019). Anal Chem (Washington, DC, U S).

[13] Backes C, Paton K R, Hanlon D, Yuan S, Katsnelson M I, Houston J, Smith R J, McCloskey D, Donegan J F, Coleman J N (2016). Nanoscale.

[14] Brennan B, Centeno A, Zurutuza A, Mack P, Paton K R, Pollard A J (2021). ACS Appl Nano Mater.

[15] Pollard A J, Brennan B, Stec H, Tyler B J, Seah M P, Gilmore I S, Roy D (2014). Appl Phys Lett.

[16] Armano A, Buscarino G, Cannas M, Gelardi F M, Giannazzo F, Schilirò E, Agnello S (2018). Carbon.

[17] Lui C H, Malard L M, Kim S, Lantz G, Laverge F E, Saito R, Heinz T F (2012). Nano Lett.

[18] Zabel J, Nair R R, Ott A, Georgiou T, Geim A K, Novoselov K S, Casiraghi C (2012). Nano Lett.

[19] Ferrari A C, Meyer J C, Scardaci V, Casiraghi C, Lazzeri M, Mauri F, Piscanec S, Jiang D, Novoselov K S, Roth S (2006). Phys Rev Lett.

[20] Nemanich R J, Solin S A (1979). Phys Rev B.

[21] Malard L M, Pimenta M A, Dresselhaus G, Dresselhaus M S (2009). Phys Rep.

[22] Havener R W, Zhuang H, Brown L, Hennig R G, Park J (2012). Nano Lett.

[23] Roscher S, Hoffmann R, Ambacher O (2019). Anal Methods.

[24] Backes C, Abdelkader A M, Alonso C, Andrieux-Ledier A, Arenal R, Azpeitia J, Balakrishnan N, Banszerus L, Barjon J, Bartali R (2020). 2D Mater.

[25] Paton K R, Varrla E, Backes C, Smith R J, Khan U, O’Neill A, Boland C, Lotya M, Istrate O M, King P (2014). Nat Mater.

[26] Backes C, Higgins T M, Kelly A, Boland C, Harvey A, Hanlon D, Coleman J N (2017). Chem Mater.

[27] Backes C, Szydłowska B M, Harvey A, Yuan S, Vega-Mayoral V, Davies B R, Zhao P-l, Hanlon D, Santos E J G, Katsnelson M I (2016). ACS Nano.

[28] Paton K R, Coleman J N (2016). Carbon.

[29] Marchesini S, Turner P, Paton K R, Reed B P, Pollard A J (2021). Nanoscale.

[30] Cançado L G, Jorio A, Pimenta M A (2007). Phys Rev B.

[31] Tuinstra F, Koenig J L (1970). J Chem Phys.

[32] Ferrari A C, Robertson J (2000). Phys Rev B.

[33] Kauling A P, Seefeldt A T, Pisoni D P, Pradeep R C, Bentini R, Oliveira R V B, Novoselov K S, Castro Neto A H (2018). Adv Mater (Weinheim, Ger).

[34] Yoon D, Moon H, Cheong H, Choi J, Choi J, Park B (2009). J Korean Phys Soc.

[35] Workman J (2010). Spectroscopy.

[36] Ciesielski A, Haar S, El Gemayel M, Yang H, Clough J, Melinte G, Gobbi M, Orgiu E, Nardi M V, Ligorio G (2014). Angew Chem, Int Ed.

[37] Haar S, Ciesielski A, Clough J, Yang H, Mazzaro R, Richard F, Conti S, Merstorf N, Cecchini M, Morandi V (2015). Small.

[38] Shin Y, Prestat E, Zhou K-G, Gorgojo P, Althumayri K, Harrison W, Budd P M, Haigh S J, Casiraghi C (2016). Carbon.

[39] Nagyte V, Kelly D J, Felten A, Picardi G, Shin Y, Alieva A, Worsley R E, Parvez K, Dehm S, Krupke R (2020). Nano Lett.

[40] Silva D L, Campos J L E, Fernandes T F D, Rocha J N, Machado L R P, Soares E M, Miquita D R, Miranda H, Rabelo C, Vilela Neto O P (2020). Carbon.

[41] Goldie S J, Bush S, Cumming J A, Coleman K S (2020). ACS Appl Nano Mater.

