# Supplementary material for: On the use of Raman spectroscopy to characterize mass-produced graphene nanoplatelets
Source: Beilstein J Nanotechnol. 2023 Apr 24;14:509–21. doi: 10.3762/bjnano.14.42 (PMC10155622; doi:10.3762/bjnano.14.42)
Supplement: File 1 — Additional experimental data. [file Beilstein_J_Nanotechnol-14-509-s001.pdf]

BEILSTEIN JOURNAL OF NANOTECHNOLOGY

Supporting Information

for

On the use of Raman spectroscopy to characterize mass-produced graphene nanoplatelets

Keith R. Paton, Konstantinos Despotelis, Naresh Kumar, Piers Turner and Andrew J. Pollard

Beilstein J. Nanotechnol. **2023,** *14,* 509–521. doi:10.3762/bjnano.14.42

Additional experimental data

License and Terms: This is a supporting information file under the terms of the Creative Commons Attribution License (https://creativecommons.org/licenses/by/4.0). Please note that the reuse, redistribution and reproduction in particular requires that the author(s) and source are credited and that individual graphics may be subject to special legal provisions.
The license is subject to the *Beilstein Journal of Nanotechnology* terms and conditions: (https://www.beilstein-journals.org/bjnano/terms)

Graphite additions

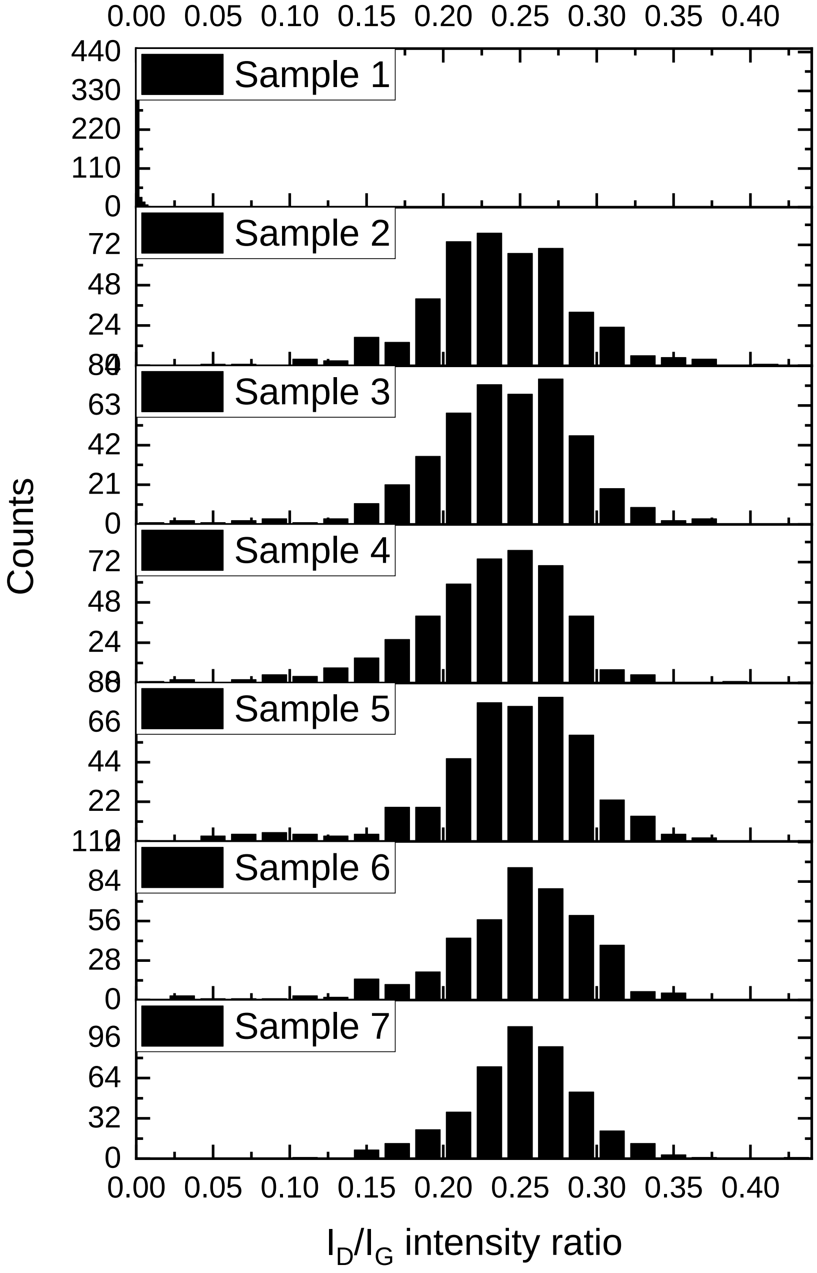

Figure S1: Histograms showing the distribution of I_D/I_G intensity ratio for samples of GNP_{ref} with graphite additions.

In the main manuscript, the values for fitted parameters were not shown for sample 7, the 100% graphite sample. Figures 3–5 are replicated here to include these values.

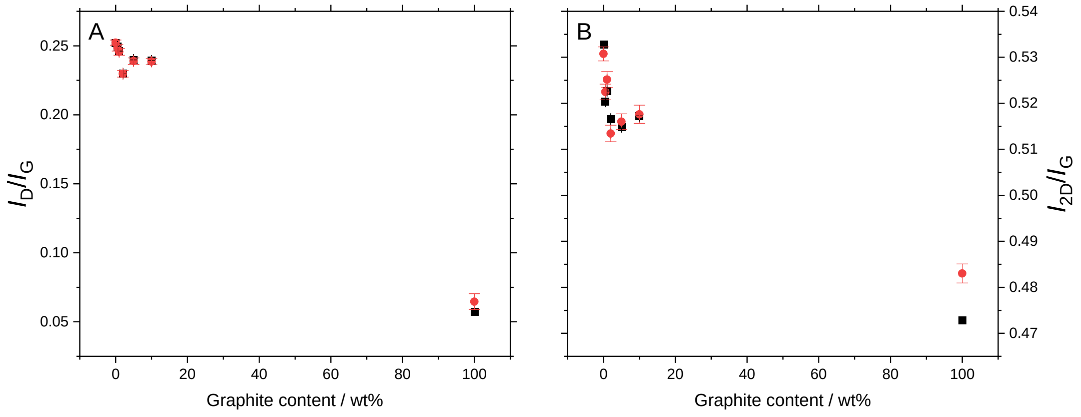

Figure S2: Fitted peak intensity ratios. (A) I_D/I_G and (B) I_{2D}/I_G, showing the values from the averaged spectra (black) with the combined standard error of the fit and the median value of the fits across each map with the standard error of the mean. Axes rescaled from Figure 3 in the main manuscript to show the 100% graphite sample.

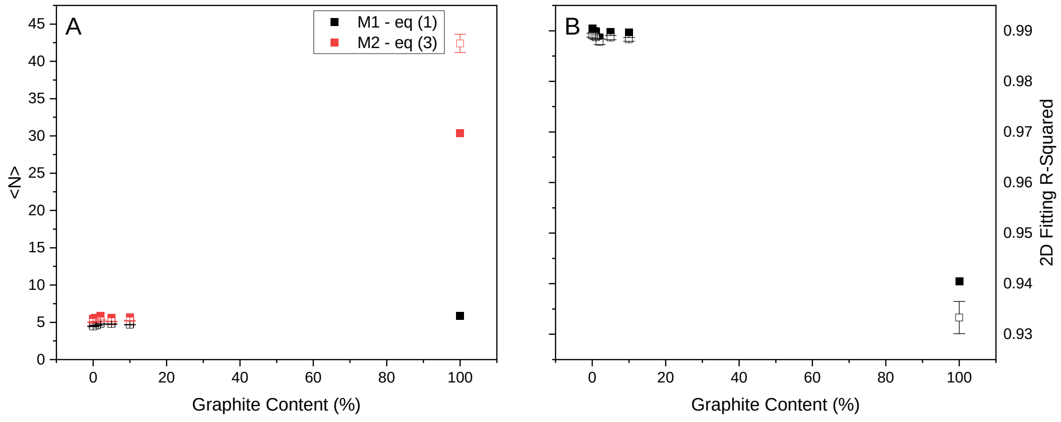

Figure S3: Comparison of literature metrics applied to the current data. (A) Mean number of layers calculated for the two metrics published by Backes and co-workers [1]. (B) R^2 value for fitting the 2D peak, as proposed by Roscher et al. [2]. In both cases, results are shown from analysis of both averaged spectra (filled markers) and individual spectra within each map (open markers). In the latter case, the median value is shown, with the error bars showing the standard deviation. Axes rescaled from Figure 5 in the main manuscript to show the 100% graphite sample.

Additional metrics

In addition to the I_D/I_G ratio, it has also been shown that the width of the G peak can provide information about the lateral size of FLG flakes in a sample [1]. Figure S4 shows the value of G peak width for samples with sediment material added, showing the median value from fitting each spectrum across the map. We note that, based on Equation 5 in Backes et al. [1], the width of the G peak measured for the FLG_{ref} sample (19.5 cm^{-1}) would predict an in-plane dimension of ca. 833 nm. This is considerably larger than the 218 nm we measured using AFM. However, we note that Backes et al. used the length of the flake, defined as the longest dimension, whereas we have used the

lateral size, as defined in ISO/TC 229 21356-1:2021(E) [3]. This will always be a smaller value than the length, as it is an average of the length and the width of the flake. While this cannot fully explain the discrepancy between the two approaches to the measurements, we also note that a recent international interlaboratory study [4] showed that the range of values for 2D peak width measured between different laboratories is between 3 and 7 cm^{-1}, based on 1.5 times the standard deviation. This range of variance could therefore explain the differences between the mean size values from the AFM measurements in this work, and the length value implied from the Raman metrics from Backes and co-workers [1].

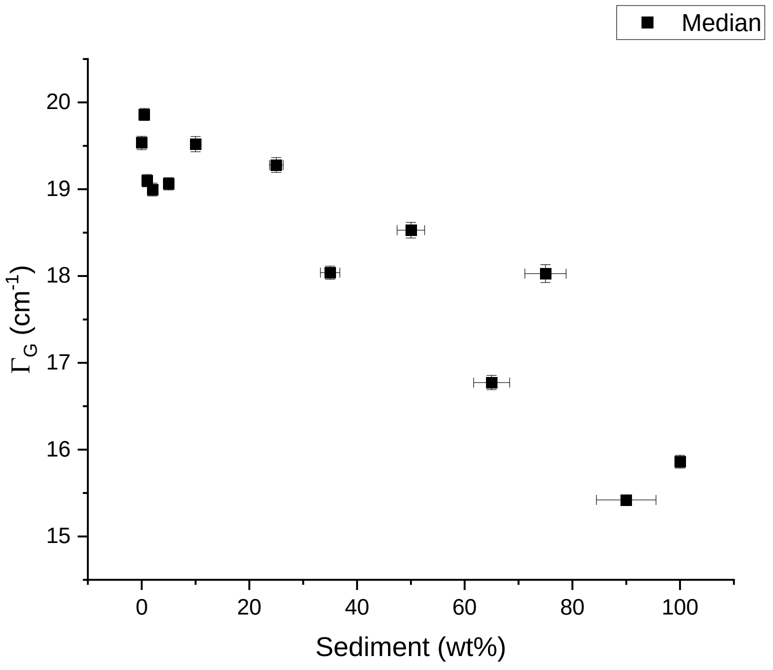

Figure S4: Median value of the width of the G peak, fitted for each spectrum across a map. The uncertainty shown is the standard error of the values.

Cançado et al. have also presented a metric to separate the effects of line defects from point defects in graphene samples [5]. This is based on the relationship between the peak area ratio A_D/A_G and the width of the G peak. We have plotted these values for the samples with sediment added (Figure S5), which confirms that the defects present in the samples are predominantly line defects, most likely flake edges. However, as for other metrics examined, it does not provide reliable identification of the presence of sediment up to ca. 25 wt %.

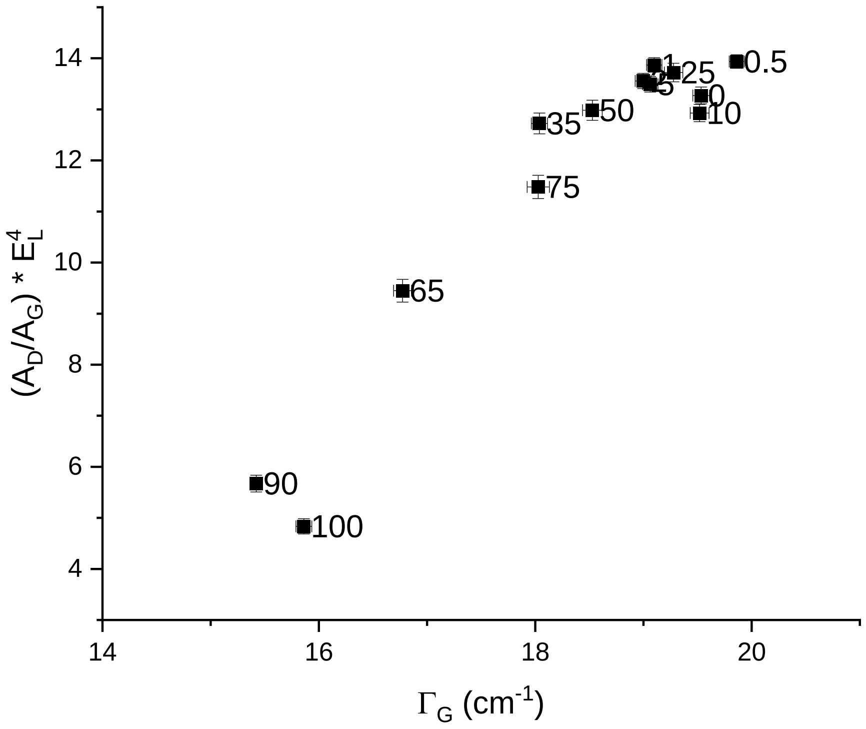

Figure S5: A_D/A_G plotted against width of the G peak (Γ_G) according to Cançado and co-workers. The values beside the data points indicate the nominal loading of sediment added to the sample.

Effect of signal-to-noise ratio on R^2-fitting

The aim of using the R^2 parameter is to characterise how well a single Voigt peak can be fitted to the data. However, it is a measure of the overall residual between the fitted function and the data. As such, when there is increased noise in the sample, the value will decrease. To investigate this, we have taken a single spectrum (from the sample with 5 wt % sediment added) and adjusted the noise level in the sample by adding a random number to each point in the spectrum. The random numbers are taken from a Gaussian distribution with a standard deviation of 0.01 to approximately match the noise level in the spectrum. This was repeated ten times. Then, increasing numbers of these spectra were averaged, and the 2D peak was fitted to a Voigt function. The quality of the fit was monitored using R^2, adjusted R^2 (which corrects for varying number of data points), and the χ^2 values (Figure S6A).

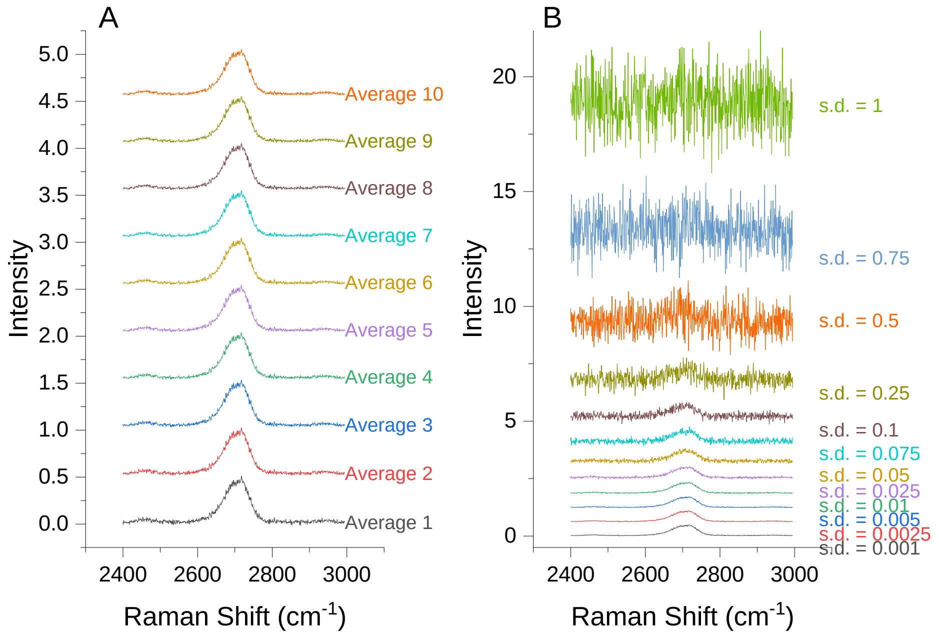

Figure S6: Spectra generated to examine the effect of the signal-to-noise ratio on the R^2 value obtained when fitting the 2D peak. Left: Averaging increasing numbers of spectra that have random Gaussian noise added to them. Right: Spectra that have increasing amounts of random Gaussian noise added.

In an alternative approach, the noise level in the same spectrum was adjusted by increasing the standard deviation of the Gaussian distribution the random numbers are selected from. The width is adjusted from 0.001 to 1, resulting in a significant change in the noise in the spectrum. The resulting spectra are shown in Figure S6B.

In both cases, the signal-to-noise (S/N) ratio is estimated by dividing the calculated area of the fitted Voigt function by the standard deviation of the data between 2800 and 2900 cm^{-1}. As seen in Figure S7, the R^2 value increases sharply as the S/N ratio increased up to about 750, exceeding the value for the original spectrum for S/N > 2500.

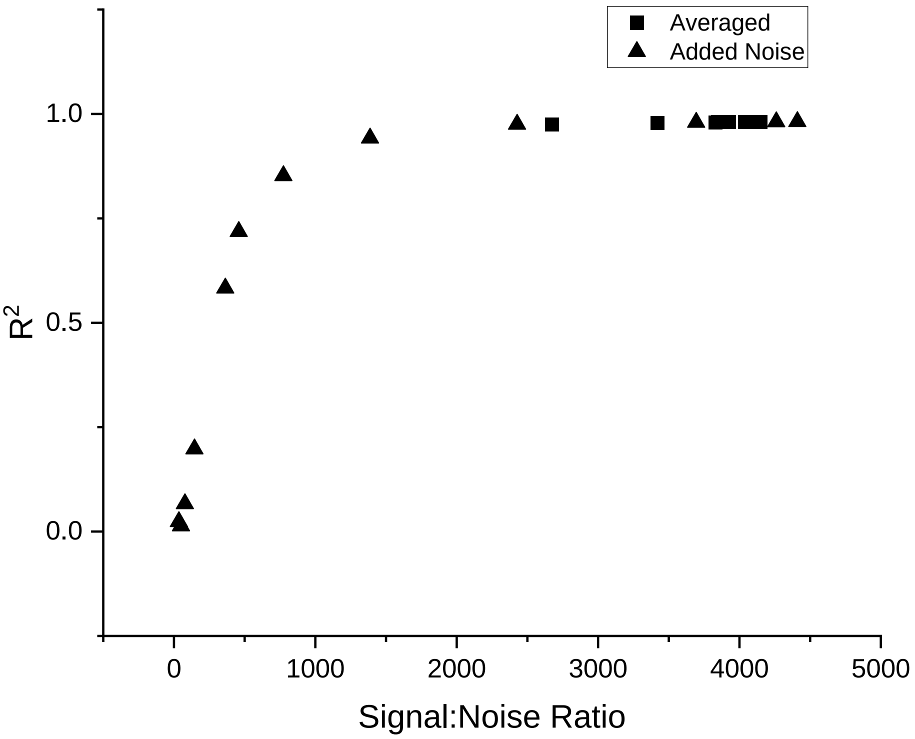

Figure S7: Value of R^2 obtained from fitting the 2D band of one spectrum to a single Voigt function as function of a signal-to-noise parameter, following the addition of artificial noise to the spectrum, as shown in Figure S4.

Fraction of sediment values

In Figure 8 of the main manuscript, values of the fraction of sediment predicted by two metrics are compared to the nominal loading. In the manuscript, the deviation from the nominal value is quantified by normalising the variance by the nominal value, as given by Equation 4. Table S1 shows values of the predicted values, normalised deviation, and ratio of predicted value to nominal.

Table S1: Value of fraction of sediment predicted from Component Analysis of Raman spectra.

Nominal loading (%)	P(Graphite) (%)	Deviation (%)	Ratio
**0**	0.907	--	0
**0.5**	1.13	129	0.436
**1**	2.72	172	0.368
**2**	1.81	9.12	1.10
**5**	2.72	45.6	1.84
**10**	5.22	47.8	1.92
**25**	3.85	84.6	6.48
**35**	5.67	84.0	6.23
**50**	9.75	80.5	5.13
**65**	22.7	65.1	2.87
**75**	20.0	73.4	3.76
**90**	49.4	45.1	1.82
**100**	57.6	42.4	1.74

Table S2: Value of fraction of sediment predicted from the R^2 value of 2D peak fitting.

Nominal loading (%)	P(Graphite) (%)	Deviation (%)	Ratio
**0**	2.04	--	0
**0.5**	1.13	129	0.436
**1**	1.36	36.0	0.735
**2**	2.27	13.6	0.880
**5**	2.49	50.1	2.01
**10**	4.76	52.4	2.10
**25**	7.26	71.0	3.44
**35**	29.7	15.9	1.19
**50**	17.2	65.5	2.90
**65**	42.9	34.1	1.52
**75**	27.2	63.7	2.76
**90**	76.4	15.1	1.18
**100**	78.9	21.1	1.27

References

1. Backes, C.; Paton, K. R.; Hanlon, D.; Yuan, S.; Katsnelson, M. I.; Houston, J.; Smith, R. J.; McCloskey, D.; Donegan, J. F.; Coleman, J. N. *Nanoscale* **2016**, *8* (7), 4311-23.doi: 10.1039/c5nr08047a
2. Roscher, S.; Hoffmann, R.; Ambacher, O. *Analytical Methods* **2019**, *11* (9), 1224-1228.doi: 10.1039/c8ay02619j
3. ISO, Nanotechnologies — Structural characterization of graphene. In *Part 1: Graphene from powders and dispersions*, 2021; Vol. ISO/TS 21356-1:2021(E).
4. Turner, P.; Paton, K. R.; Legge, E. J.; de Luna Bugallo, A.; Rocha-Robledo, A. K. S.; Zahab, A.-A.; Centeno, A.; Sacco, A.; Pesquera, A.; Zurutuza, A.; Rossi, A. M.; Tran, D. N. H.; L Silva, D.; Losic, D.; Farivar, F.; Kerdoncuff, H.; Kwon, H.; Pirart, J.; Campos, J. L. E.; Subhedar, K. M.; Tay, L.-L.; Ren, L.; Cançado, L. G.; Paillet, M.; Finnie, P.; Yap, P. L.; Arenal, R.; Dhakate, S. R.; Wood, S.; Jiménez-Sandoval, S.; Batten, T.; Nagyte, V.; Yao, Y.; Hight Walker, A. R.; Martins Ferreira, E. H.; Casiraghi, C.; Pollard, A. J. *2D Materials* **2022**, *9* (3), 035010.doi: 10.1088/2053-1583/ac6cf3
5. Cançado, L. G.; Kampioti, K.; Huang, K.; Silva, M. G. d.; Pénicaud, A.; Ferreira, E. H. M.; Achete, C. A.; Hof, F.; Capaz, R. B.; Jorio, A. *2D Materials* **2017**, *4*.doi: 10.1088/2053-1583/aa5e77
